# Synaptic Plasticity Enables Adaptive Self-Tuning Critical Networks

**DOI:** 10.1371/journal.pcbi.1004043

**Published:** 2015-01-15

**Authors:** Nigel Stepp, Dietmar Plenz, Narayan Srinivasa

**Affiliations:** 1 Center for Neural and Emergent Systems, Information and System Sciences Lab, HRL Laboratories LLC, Malibu, California, United States of America; 2 Section on Critical Brain Dynamics, Laboratory of Systems Neuroscience, National Institutes of Mental Health, Bethesda, Maryland, United States of America; Philipps-University Marburg, GERMANY

## Abstract

During rest, the mammalian cortex displays spontaneous neural activity. Spiking of single neurons during rest has been described as irregular and asynchronous. In contrast, recent in vivo and in vitro population measures of spontaneous activity, using the LFP, EEG, MEG or fMRI suggest that the default state of the cortex is critical, manifested by spontaneous, scale-invariant, cascades of activity known as neuronal avalanches. Criticality keeps a network poised for optimal information processing, but this view seems to be difficult to reconcile with apparently irregular single neuron spiking. Here, we simulate a 10,000 neuron, deterministic, plastic network of spiking neurons. We show that a combination of short- and long-term synaptic plasticity enables these networks to exhibit criticality in the face of intrinsic, i.e. self-sustained, asynchronous spiking. Brief external perturbations lead to adaptive, long-term modification of intrinsic network connectivity through long-term excitatory plasticity, whereas long-term inhibitory plasticity enables rapid self-tuning of the network back to a critical state. The critical state is characterized by a branching parameter oscillating around unity, a critical exponent close to -3/2 and a long tail distribution of a self-similarity parameter between 0.5 and 1.

## Author Summary

Neural networks, whether artificial or biological, consist of individual units connected together that mutually send and receive parcels of energy called spikes. While simply described, there is a vast space of possible implementations, instantiations, and varieties of neural networks. Some of these networks are critically balanced between randomness and order, and between death by decay and death by explosion. Selecting just the right properties and parameters for a particular network to reach this critical state can be difficult and time-consuming. The strength of connections between units may change over time via synaptic plasticity, and we exploit this mechanism to create a network that self-tunes to criticality. More specifically, the interplay of opposing forces from excitatory and inhibitory plasticity create a balance that allows self-tuning to take place. This self-tuning takes relatively simple spiking units and connects them in a way that creates complex behavior. Our results have implications for the design of artificial neural networks implemented in hardware, where parameter tuning can be costly, but may provide insight into the critical nature of biological networks as well.

## Introduction

The mammalian cortex presents a challenging complex system for the study of information processing, behavioral adaptation, and self-organization. At rest, a state in which there is no obvious sensory input or motor output, neural activity in the cortex is predominantly spontaneous, or ongoing. At the single neuron level, resting activity has been characterized as persistent and irregular firing of action potentials, or spikes. A well-known aspect of cortical spiking is that, at rest, the correlation between distant, single neuron spiking is very low [1]. Persistent asynchronous background activity (PABA), however, is typically interpreted as a largely independent activity. Independence does not seem concomitant with the cortex as a complex system, which typically displays interactions among most system elements and long-range structure as detailed below. Demonstrations regarding the exquisitely high sensitivity of cortical networks to the addition of even a single spike [2] have further fueled the debate concerning robust cortical computation in the presence of apparently uncorrelated contributions from single neurons [2, 3].

Other research, however, has demonstrated that spontaneous cortical activity *in vitro* [4–6] and *in vivo* [1, 7, 8] at the population level manifests as precisely organized spatiotemporal cascades of activity termed neuronal avalanches. For critical networks, the scale-invariance of avalanche sizes is reflected by a power-law with exponent −3/2. Such a power-law is expected when cortical networks are balanced so that spiking activity neither tends to increase nor decrease, a state quantified by the critical branching ratio *σ* = 1 [4].

Theory predicts that networks with critical dynamics optimize numerous aspects of information processing [9]. Specifically, experiment and modeling show maximized information capacity and transmission [5], maximized number of metastable states [10, 11], optimized dynamic range [12, 13], and optimum variability of phase synchrony [6]. The ubiquity of scale-invariance in nature, combined with its advantages for information processing, suggests that each of the foregoing properties would be beneficial for neuronal models and artificial systems, i.e. physical embodiments of neuronal networks, as well [14, 15]. In all of these cases, the networks in question are *critical* and not merely *balanced*.

Recently, both conservative [16] and non-conservative [17] neural networks featuring short-term synaptic plasticity (STP) have been demonstrated to be critical, or display neuronal avalanches. Likewise, neural networks that incorporate long-term synaptic plasticity, such as spike-timing dependent plasticity (STDP), have displayed balanced networks as well [18, 19]. These models, however, did not exhibit self-generated PABA [3, 20] and were not capable of self-tuning to criticality after being steered away from it by strong perturbations. A neural network that was capable of self-tuning to stable regimes based on short-term plasticity was described in [21], however that network did not exhibit critical dynamics and did not include long-term STDP that can create lasting changes to synaptic conductances. We show that it is possible for a *single* neural network to exhibit four of the above properties *simultaneously*, namely PABA, self-tuning due to short-term plasticity (as in [16, 17]) and long-term plasticity (as in [18–19, 22]) and critical balance (as in [16, 17]). We also show that such a network undergoes a lasting change in synaptic strengths, thereby effective connectivity, suggesting the capability of learning. Combining all of these properties into a single system greatly diminishes the need for external controls in order to establish the desirable network dynamics and behavior.

To that end, we demonstrate in a 10,000 neuron network model with 20% inhibitory neurons including AMPA and GABA-receptor dynamics, how a network self-tunes to criticality. This deterministic network spontaneously displays PABA and undergoes changes in network dynamics and structure due to short- and long-term synaptic plasticity in the response to perturbations, while self-tuning back to a critical regime. We believe that this capability will lead to the realization of future synthetic physical systems that self-tune to be optimally sensitive in response to multi-scaled stimuli and adapt to changing environmental conditions, thus paving the way for synthetic intelligent systems [14–15, 23, 24].

## Results

### Network spiking behavior

A 10,000 neuron network with both excitatory (80%) and inhibitory (20%) neurons was simulated for 900 s in 1 ms time-steps. The simulator itself [25] was based on the dynamics and feature set of a specific neuromorphic hardware implementation [15, 26]. These features are short term plasticity (STP), spike-timing dependent plasticity (STDP), and AMPA and GABA-receptor kinetics. For details of the network model and simulation parameters see Materials and Methods.

The simulation began by injecting Poisson-distributed spikes at a 300 Hz firing rate into a randomly chosen set of 20 excitatory (E) neurons. After 15 ms, the initializing external drive ceased and the network was left to develop its own internal dynamics. The effect of varying these initial perturbation parameters is not well known, and is a subject open for further study. After the initial 15 ms, the network stabilized to a spontaneous firing mode where it maintained an average firing rate of 31.8 ± 2.6 Hz. Average rate is defined as the number of spikes produced by the network per unit time, divided by the number of neurons.

**Figure 1:**
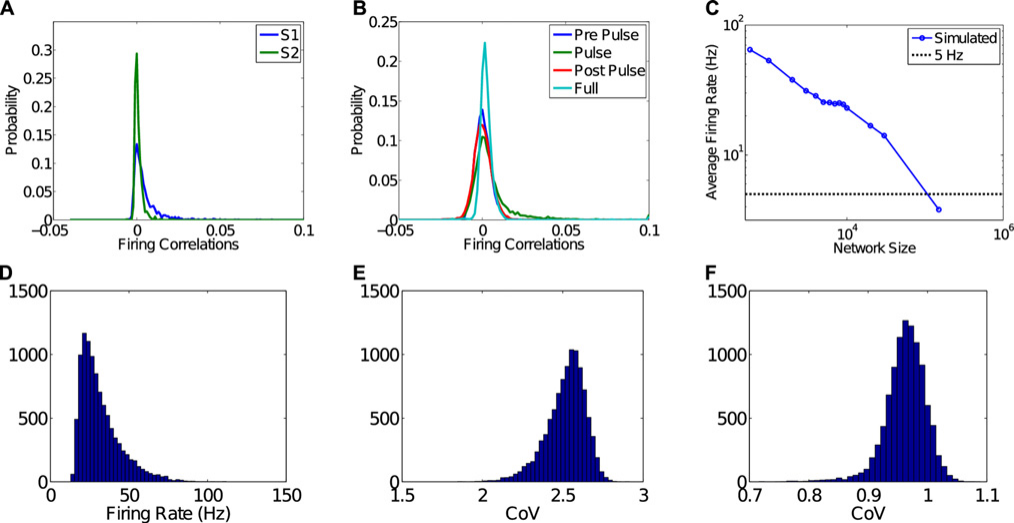
Neurons are uncorrelated during PABA and exhibit Poisson-like irregularity. A) Neuronal firing is weakly correlated during ongoing activity in awake monkeys that display neuronal avalanches in the local field potential (LFP) Distributions of pair-wise correlation coefficients of firing in the pre-motor cortex of two awake monkeys, S1 (1.2 Hz mean rate) and S2 (5.2 Hz mean rate). For details see the Materials and Methods section and [1, 27]. B) Distributions of pair-wise correlation coefficients for 50 excitatory and 50 inhibitory simulated spike-trains from 200 s to 300 s (pre-pulse), 300 s to 400 s (during pulse), 800 s to 900 s (post-pulse), and 20 s to 900 s (full). All distributions are centered close to or at 0 demonstrating uncorrelated firing. In addition, if responses to inputs are included (see below) (during pulse and full), the distributions are slightly right-tailed reflecting the increase in correlation due to common input. C) Dependence of firing rate on network size. As the number of neurons increases, the average firing rate decreases. At sizes above 100,000 neurons, i.e. on the order of a small collection of cortical columns, firing rates drop to below 5 Hz, which is comparable to biological networks. Each data point represents the results of a parameter search at that network size, minimizing firing rate. D) Distribution of firing rates in the network. E) Coefficient of Variation for ISIs between 3 ms and 1000 ms. F) Coefficient of Variation for ISIs between 10 ms and 1000 ms. The *CoV* plots show that including ISIs between 3 ms and 10 ms greatly affects the measured irregularity of spiking intervals, possibly suggesting a high-frequency bursting process.

Spiking was asynchronous and irregular as quantified by both pairwise correlations between spike-trains and the coefficient of variation of inter-spike-intervals. Specifically, pairwise correlation distributions from spontaneous activity in the model were centered around 0, as shown in Fig. 1b. Such weak correlation in spiking in our model is in line with the weak pairwise correlations in spiking found in ongoing activity of awake monkeys that demonstrate neuronal avalanche organization in the local field potential [1, 27] (Fig. 1a). They are also similar to near zero-mean correlations found for spiking *in vivo* in both rats and monkeys, and simulations of large balanced networks that do not exhibit continuous synaptic plasticity [28, 29]. While the firing rate achieved in our model is higher than observed in mammalian neuronal networks [3, 20], we find that as network size increases, firing rate decreases to below 5 Hz for networks larger than 100,000 neurons (Fig. 1c).

In order to quantify the irregularity of spiking in the network, we calculated distributions of the coefficient of variation, $CoV=\frac{\sigma }{\mu }$, of inter-spike-intervals (ISIs). That is, the ratio of the standard deviation of a series of ISIs to the mean ISI. If the standard deviation is greater than the mean, i.e. if *CoV* ≥ 1, the ISIs are considered irregular. Fig. 1d shows the distribution of firing rates for the network. For a range of ISIs between 3 ms and 1000 ms, equivalent to a range between 300 Hz and 1 Hz, the distribution of *CoV* is as shown in Fig. 1e. This *CoV* distribution is heavily skewed and centers near 2.5, demonstrating highly irregular spiking. If the range of ISIs is restricted further to those between 10 ms and 1000 ms (100 Hz to 1 Hz) (Fig. 1f), then the distribution of *CoV* peaks slightly below unity, suggesting an exponential distribution of ISIs close to that expected from a Poisson process. Thus, a considerable contribution to spike irregularity originates from action potential bursts at 100–300 Hz.

**Figure 2:**
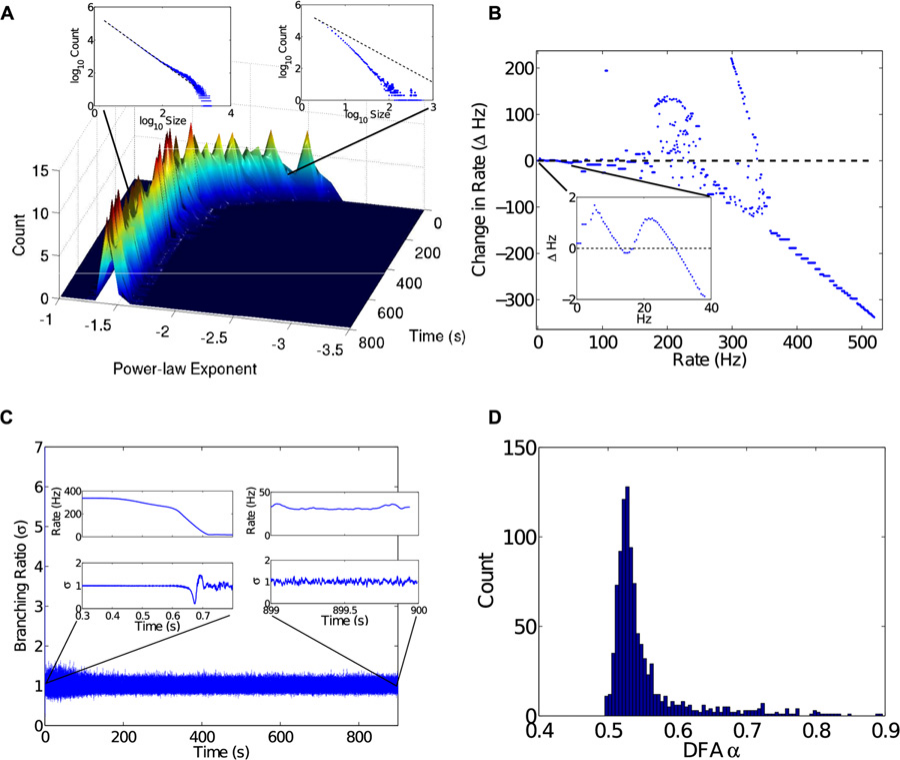
Criticality analyses. A) The exponent *λ* was measured during network evolution for a sliding time-window, then further grouped into a sliding window of *λ* estimates in order to show histograms changing over time. The top right inset shows sub-critical behavior (*t* = 20 s, *λ* = −2.49, *R* ^2^ = 0.9855, *p* < 0.001). Once the network reaches a steady state exhibiting PABA, the network becomes critical with *λ* ≈ −1.5. For example, the late stage inset (top left) shows critical behavior (*t* = 500 s, *λ* = −1.481, *R* ^2^ = 0.9984, *p* < 0.001). B) Estimated deterministic dynamics for the firing rate of the network. Data for firing rates over 50 Hz come entirely from the first 500 ms of the simulation, where the network goes through a period of stabilization. Firing rates during that period visit a sequence of multi- and meta-stable states until settling into a stable fixed point near 30 Hz (inset). C) The branching ratio *σ*. The two insets show *σ* at different stages. The early stage shows a very high firing rate regime, and a steep drop to a relatively low firing rate during which *σ* fluctuates widely. Eventually the network settles down, at about 0.7 s, and oscillates around *σ* = 1 for the remainder of the simulation. D) The distribution of scaling exponents *α* measured from 1 s to 900 s for 1000 randomly chosen neurons. All exponents lie between 0.5 and 1.0, indicating correlated fluctuations.

Treating the firing rate of the network as a dynamical system in its own right, it is possible to estimate fixed points in order to identify stable (and unstable) firing rates. Assuming a Langevin model for the firing rate, Fig. 2b shows a reconstruction of the deterministic dynamics [30, 31]. Firing rates greater than 50 Hz exist entirely in the first 500 ms of the simulation, when the network goes through a stabilization period. During this period, firing rates visit a sequence of multi- and meta-stable states until settling into a stable fixed point near 30 Hz. More study is required to know whether the fixed points at greater firing rates still exist or have been annihilated due to bifurcations, a bifurcation being a change in the number or type of fixed points. It is worth noting that a stable and unstable fixed point pair near 10 Hz (see Fig. 2b inset) is very near a bifurcation.

### Network criticality

Even though the spiking behavior of the network is classified as uncorrelated, i.e. asynchronous and irregular, there might still be identifiable structure. Specifically, causal spikes, or spikes that cause other spikes, can be grouped into avalanches of particular sizes; the inflation or deflation of causal spikes as they propagate through the network can be balanced or unbalanced; and fluctuations in ISI can be correlated or uncorrelated. If these three measures take on particular values, the network is said to be in a critical state, which we detail below.

The first measure is based on avalanche sizes, where an avalanche is identified as a set of contiguous spiking events. If a neuron spikes without any input from a presynaptic neuron, it is the beginning of an avalanche. If a neuron spikes due to incoming spikes, the new spike is a member of the same avalanche as the spiking presynaptic neurons. In a causally closed network, this definition only makes sense for a subgraph of the network, where inputs that start avalanches are allowed to come from neurons outside of the subgraph.

Avalanche size is defined as the number of spikes that belong to an avalanche, and the distribution of sizes is particular to the properties of the network. If this distribution follows a power-law, it will produce a straight line when plotted on a log-log scale. The slope of this line, *λ*, is related to the power-law exponent.

It has been observed, both experimentally and following from theory [4], that neuronal networks behaving in the critical regime have *λ* = −3/2. Using an avalanche tracking algorithm (see Methods), we measured the avalanche size distribution for a sliding 100 s window, producing estimates for *λ* over time, which were further grouped into sliding windows of 20 *λ* estimates to produce histograms over time (Fig. 2a). After about 300 s into the simulation, *λ* ≈ −3/2. In our analysis, 12.5% of neurons were randomly sampled by the avalanche tracking algorithm (see Methods). Random sampling produces a relatively normal distribution of *λ* estimates. After 500 samples for the period of time between 500 s and 600 s, the distribution of corresponding *λ* estimates had a mean of -1.620 with standard deviation 0.0700.

The second measure assessed the branching ratio *σ* of the network. The branching ratio *σ* measures the average ratio of postsynaptic spikes to presynaptic spikes. If *σ* < 1, or is sub-critical, the spiking activity in the network decays. If *σ* > 1, or is super-critical, then spiking activity in the network grows. A branching parameter *σ* = 1 signifies a stationary network where, on average, the number of spikes received by a neuron results in about the same number of spikes emitted by postsynaptic neurons [32]. The branching ratio over the course of the simulation was measured and is plotted in Fig. 2c. Oscillations of *σ* about unity indicate that the network is stable in the context of critical branching. Such stability is a necessary, but not sufficient, condition for the more subtle property of criticality as measured by avalanche size distributions above.

As a third measure of criticality, the network simulation was analyzed using detrended fluctuation analysis (DFA) [8, 33], which measures how the variance of fluctuations in spiking activity changes over changes in measurement scale (see Methods). DFA estimates a scaling exponent *α*, which, when in the range 0.5 < *α* < 1, indicates positive long-range correlations in the fluctuations. Results of DFA for individual spike-trains are presented in Fig. 2d as a distribution of scaling exponents with mean *α* = 0.68 (*SD* = 0.061). This distribution shows that scaling exponents are spread among the range that coincides with correlated fluctuations.

#### Critical networks are finely balanced

**Figure 3:**
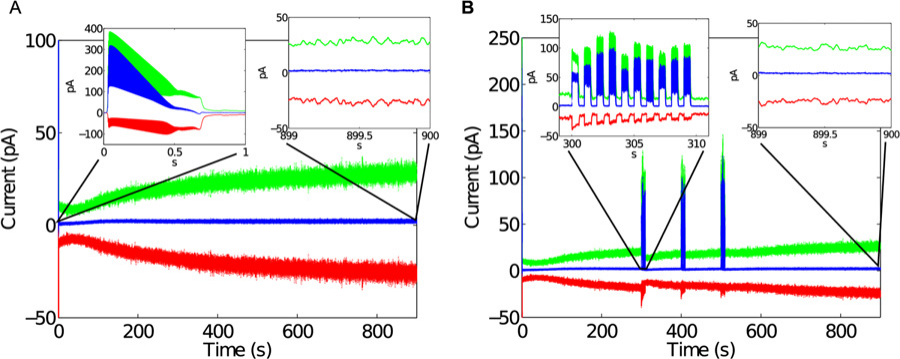
Balance between excitatory and inhibitory currents during stable expression of PABA. A) During the initial 0.7 s when the network dynamics evolves towards lower firing rates, the network is not balanced as shown by E (green) and I (red) synaptic currents and net synaptic current (blue). Balance in E/I develops after about 0.7 s at lower firing rate even when the network is not yet critical (cf. Fig. 2a). As the overall strength in E/I currents increases, the network establishes critical dynamics (about 200 s, cf. Fig. 2a). B) The net synaptic current shows a balance between *E* and *I* synaptic currents for the perturbed case despite brief, temporary imbalances due to the perturbations.

As alluded to above, critical networks, and critical systems in general, are so called because they are balanced between two otherwise stable states or behaviors. Avalanche size distributions, branching ratios, and scaling exponents are designed to test properties of criticality, but it is possible to verify the balance question directly. First, the currents from excitatory and inhibitory synapses were separated and tallied. Fig. 3 shows excitatory and inhibitory currents, as well as their difference. The small difference between both currents demonstrates that excitation and inhibition are in a state of balance. Strong perturbations, described below, caused the balance to be offset, but balance was regained after perturbations ceased.

**Figure 4:**
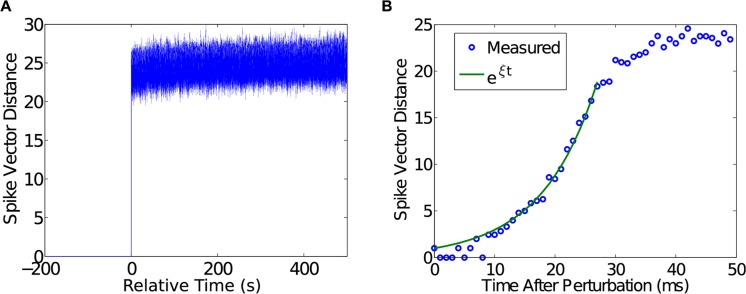
Divergence due to a single spike perturbation. A) Spike vector distance at each time-step between a control simulation without perturbations and an otherwise identical simulation with a single extra spike introduced at *t* = 300 s. The *x*-axis is plotted in time relative to the introduction of the single spike. B) Divergence of spike vector distance follows an exponential function (exp*ξt*, *ξ* = 0.1087 ± 0.0017, 95%*CI* = (0.1070, 0.1104), *R* ^2^ = 0.9782) until it reaches a saturation point that is near the expected distance between two random spike vectors.

The maintenance of balance suggests an active mechanism, the sensitivity of which can be probed by injecting a single spike into the simulation. At 400 s into the simulation, a single neuron was made to spike when it would not have ordinarily. The network was otherwise unperturbed. Since our network evolves deterministically, it is possible to directly compare the evolution of its activity in the presence and absence of the single-spike perturbation. The corresponding spike vector differences between the perturbed and unperturbed cases are shown in Fig. 4. As can be seen in this figure, the addition of a single spike leads to a significant spike vector difference of about 25 spikes, which remains approximately constant throughout the remainder of the simulation. At high temporal resolution (Fig. 4b), the single spike initiates an exponential departure from the unperturbed state over the course of about 40 ms before a new, stable state is reached.

### Self-tuning and the role of synaptic plasticity

A network could be balanced, or positioned, at a critical state by, among other possibilities, the adjustment of network parameters or input properties. In order for a network to seek criticality, instead of merely being positioned there, it must be able to alter itself. The ability of the simulated network to alter itself was tested by subjecting a subset of excitatory neurons to externally applied perturbations. Perturbations were organized into a series of 10 pulses of 300 Hz spiking, each lasting 500 ms and separated by 500 ms of silence. Three such perturbations were applied, at 300, 400, and 500 s, respectively.

**Figure 5:**
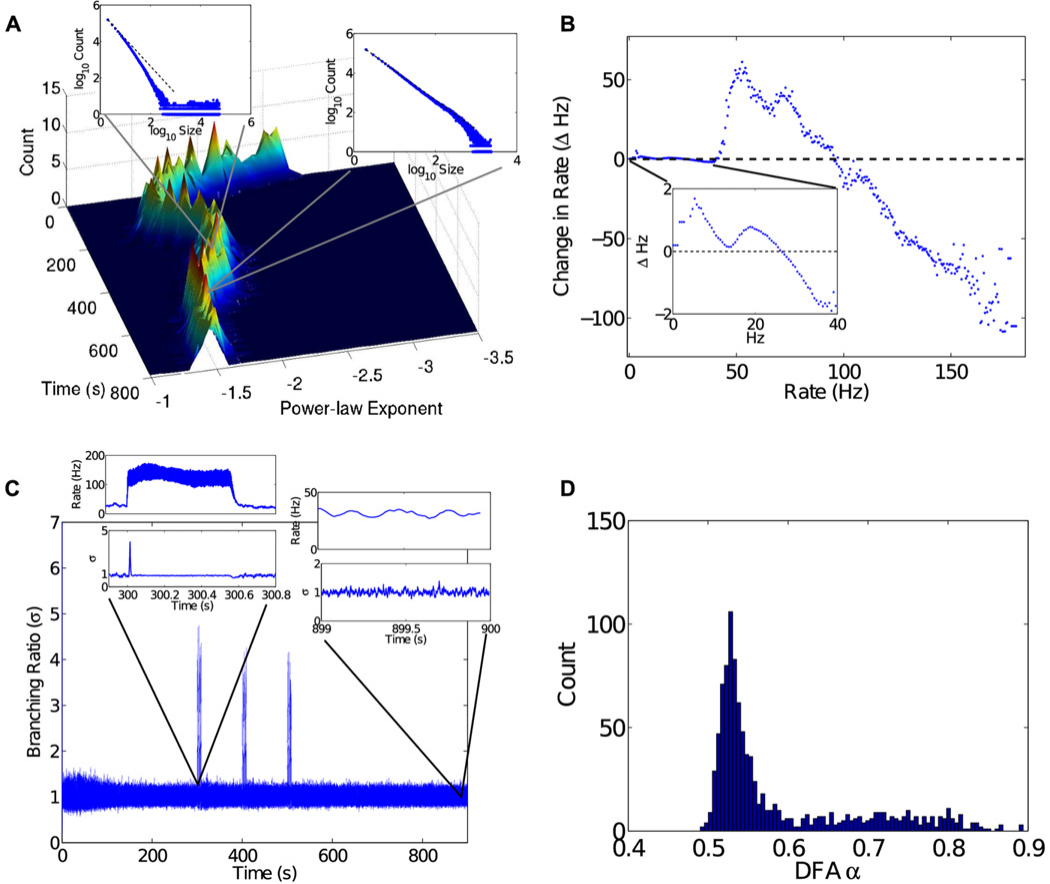
Criticality analyses showing the effect of strong perturbations. A) The power law exponent *λ* was estimated during network evolution for a sliding time-window, then further grouped into a sliding window of *λ* estimates in order to show histograms changing over time. The network settles into critical behavior with PABA as shown in the top left inset (*t* = 200 s, *λ* = −1.5415, *R* ^2^ = 0.9998, *p* < 0.001). During the perturbation the network goes into a super-critical regime as shown in the second inset (*t* = 500 s, *λ* = −1.8851, *R* ^2^ = 0.9967, *p* < 0.001) with *λ* for the initial part of the distribution between −1.75 and −2, but with a concentration of larger avalanches. The network returns to criticality after the perturbation as in the bottom right inset (*t* = 600*s*, *λ* = −1.5592, *R* ^2^ = 0.9991, *p* < 0.001). B) Estimated deterministic dynamics for the firing rate of the network. Data for firing rates over 150 Hz come entirely from the first 500 ms of the simulation (see Fig. 2b), where the network goes through a period of stabilization. Rates between 50 and 150 Hz are due to external perturbation, with an evident fixed-point near 100Hz. Otherwise, the network settles into a fixed point near 30 Hz (inset). C) The branching ratio *σ*. The two insets show *σ* during a perturbation and after recovery. The latter inset should be compared to Fig. 2c, to see that branching ratio dynamics return to the same qualitative state. D) The distribution of scaling exponents *α* for 1000 neurons. All exponents lie between 0.5 and 1.0, indicating correlated fluctuations. The bulge in the tail of the distribution for 0.6 < *α* < 0.85 is due to the change in structural connectivity caused by STDP after the three perturbations.

The three criticality analyses were repeated to confirm that criticality was reattained after such perturbations. These three measures, *λ*, *σ*, and *α* are shown for the perturbed simulation in Fig. 5. Together, they show that the network did indeed reattain criticality. As a point of interest, the deterministic dynamics of the network firing rate were again reconstructed, this time showing the dynamics present during external perturbation. The stable point reached during a perturbation is visible as a stable fixed point near 100 Hz. Notably, the perturbations have appeared to push the low firing rate fixed point near 10 Hz through a bifurcation (see Fig. 5b inset). Care must be taken with this interpretation, however, as the data only represent an approximation of deterministic dynamics.

We may also note the effect of constant random perturbations. Such input to the network would provide starting points for new avalanches throughout the whole simulation. Providing a constant 1 Hz input of Poisson-distributed spikes causes the network to tune towards criticality faster (possibly due to increased activity of STDP), but does not alter avalanche size distributions. Another method of disturbing the network is to add variation to the network parameters in Table 1. We added 1% noise to each parameter throughout a simulation and observed that self-tuning to criticality was still manifested.

**Table 1:** The eight parameters for the candidate network obtained from a parameter search to identify neuronal networks that exhibit PABA, critical behavior and self-tuning to criticality.

Parameter	Description	Value	
*A* ^+^	Max value for change in potentiation for E-STDP	0.0015	
*β*	Ratio of area under E-STDP curve for depression over potentiation	1.21	
$g_{exc}^{max}$	Max synaptic conductance for *E* → *E* and *E* → *I* synapses	1.94	nS
$g_{inh}^{max}$	Max synaptic conductance for *I* → *E* and *I* → *I* synapses	4.74	nS
*τ*_*AMPA*_	Time constant for AMPA kinetics	19	ms
*τ*_*GABA*_	Time constant for GABA kinetics	14	ms
*τ*_*F*_	STP facilitation time constant	41	ms
*τ*_*D*_	STP depression time constant	26	ms

**Figure 6:**
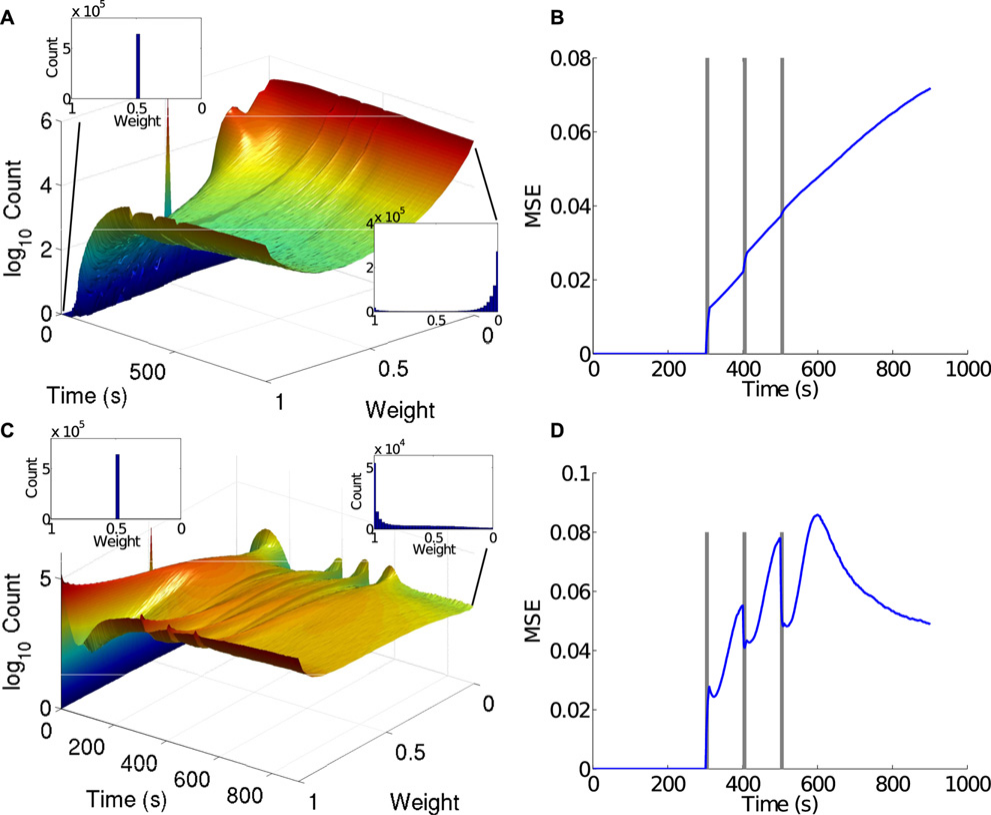
Evolution of synaptic conductances in the network. A) The evolution of *E* synapses in the *E*–*I* network shows that conductances develop a bimodal distribution, heavily favoring weights close to zero. The initial *E* synaptic conductances were set to 0.5 nS for all synapses. B) The mean square error (MSE) between *E* synaptic conductances with and without perturbations during network evolution. Each perturbation causes a jump in weight differences, with weights continuing to diverge between perturbations. C) The evolution of *I* synapses in the *E*–*I* network shows that the synaptic weights develop a unimodal, and qualitatively exponential, distribution of synaptic conductances. The initial *I* synaptic conductances were set at 0.5 nS for all synapses. D) The MSE between *I* synaptic conductance histograms with and without perturbations. Inhibitory weights tend to recover from perturbations more than excitatory weights.

As argued above, self-tuning to criticality requires change within the network, which is most readily effected by altering synaptic conductances. Fig. 6 shows the changing distribution of these synaptic “weights” over the course of the simulation. While the effect of perturbations on E and I synaptic weights is evident visually, by the three ridges in the weight-time plot respectively (Fig. 6a,c) it can be further quantified by comparing the perturbed and unperturbed weights using a simple mean square error measure (see Methods). This measure shows that each perturbation caused strong change to the synaptic conductances in both E and I weights, which outlasted the perturbation. Thus, these perturbations also significantly changed the effective network topology as well.

**Figure 7:**
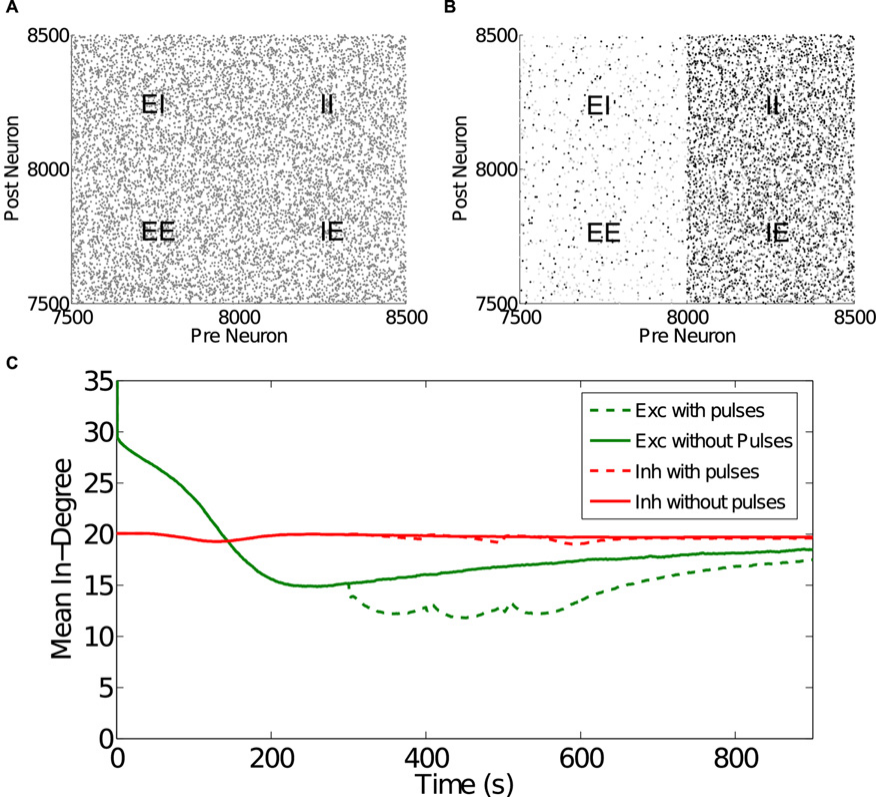
Plot of graph matrices for a subset of neurons at A) 0 s simulation time and B) 300 s simulation time. Each dot represents a synaptic weight on a linear scale such that white and black correspond to 0 and 1, respectively. At 0 s connections are distributed randomly with all weights set to 0.5. After 300 s, excitatory weights have mostly dropped towards 0, with just a few weights increasing towards 1. Inhibitory weights increased towards 1 with only a few connections being reduced. C) The time-series of mean in-degree for excitatory and inhibitory synapses in both pulsed and non-pulsed cases. Note that excitatory in-degree is approximately 80 at time 0 s, but drops dramatically during the first second.

Speaking more directly to topology, it is possible to define an in-degree, the number of incoming connections to a neuron, using a synaptic weight threshold. In this manner, a connection was considered present if its strength had a value of at least 0.1. Applying this threshold, the unperturbed simulated network began (Fig. 7a) with a mean excitatory in-degree of 80.07 (*SD* = 8.862) and a mean inhibitory in-degree of 20.06 (*SD* = 4.471). Since pre and post neurons for all connections were chosen using the same random selection procedure, in- and out-degrees were approximately equal. After 300 s of simulation time (Fig. 7b) the mean in-degree of excitatory synapses dropped significantly to 15.19 (*SD* = 3.845, *t*(15998) = 600.7, *p* < 0.001). Inhibitory in-degree remained largely unchanged at 19.93 (*SD* = 4.341, *t*(15998) = 1.901, *p* = 0.057). Fig. 7c shows the in-degree time-series for both perturbed and unperturbed cases.

**Figure 8:**
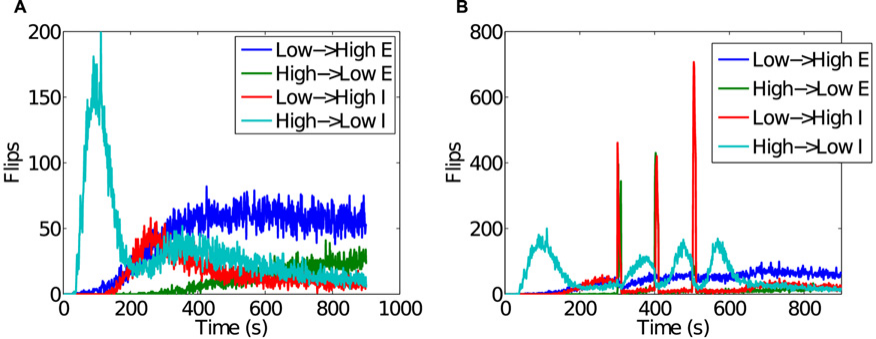
Occurrences of a synaptic weight either transitioning from low (< 0.1) to high (> 0.9) or *vice versa*. A) Transitions during an unperturbed simulation. B) Transitions during a perturbed simulation.

It is unclear from the relatively stable mean in-degree depicted in Fig. 7c whether the degree of connectivity between neurons is statically or dynamically stable. That is, the stability of the mean could be due to slowly changing connectivity, or connectivity could be changing rapidly while maintaining a near constant mean value. This question was addressed by examining which and how often synapses transitioned between strong and weak (see Methods). The occurrence of these so-called “flips” is summarized in Fig. 8, showing that connectivity is most likely the result of rapidly changing synapses that combine to a mean value that changes over a slower time-scale.

#### Inhibitory plasticity

**Figure 9:**
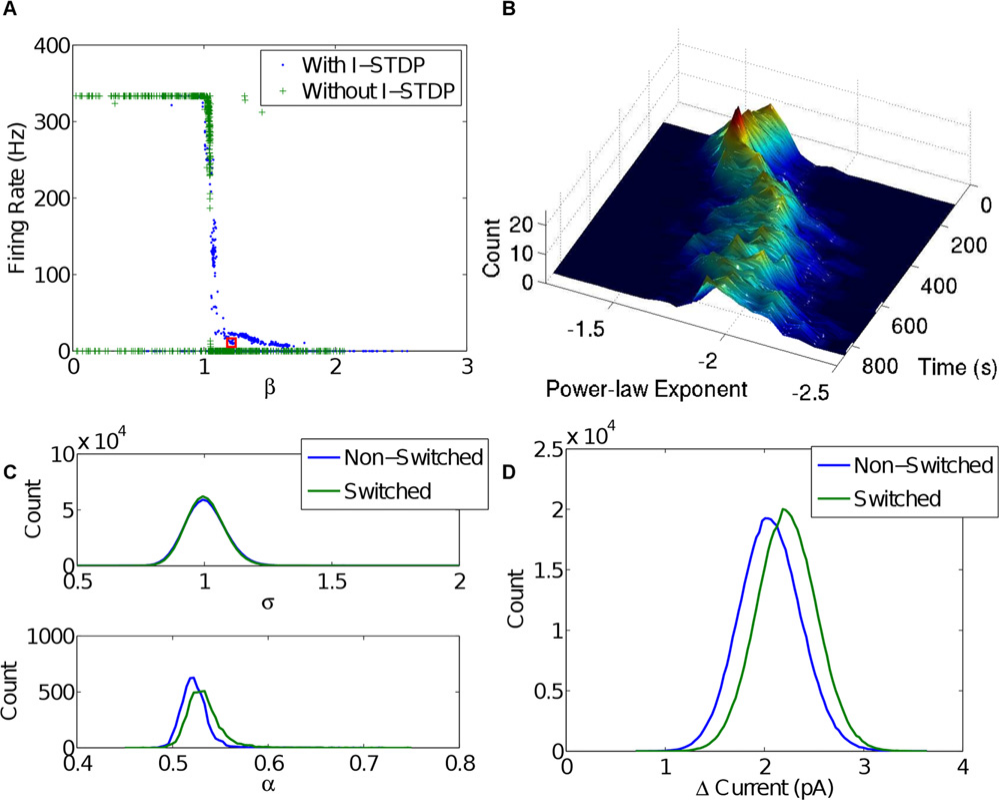
Dependence of criticality and balance on Inhibitory STDP. A) The relationship between *β* and firing rate found for many simulation instances during a parameter search. The red square indicates the network described in Table 1. If inhibitory STDP is included, there is a region *β* ≈ [1.1,1.7] where relatively low firing rates are possible. Without inhibitory STDP, there is a critical value at *β* ≈ 1.05 that separates extremely high firing rates from networks that do not persist. B) A demonstration that if inhibitory STDP is turned off at 300 s, the avalanche power-law exponents fail to tune towards −1.5. Compare with Fig. 2a. C) The distribution of branching ratio over time and DFA *α* over neurons after the switch at 300 s. These show a decoupling of balance and criticality. D) Distributions of current balance after the switch at 300 s, showing that balance is retained even after I-STDP is switched off.

Plasticity of inhibitory synaptic connections is supported by recent experimental and theoretical findings [22, 34]. To judge the importance of inhibitory plasticity, the network was simulated both with and without inhibitory STDP. Fig. 9a shows firing rates achieved as a function of one of the varying network parameters, *β* (the E-STDP depression to potentiation ratio). In the presence of inhibitory STDP, there is a reasonably wide range of *β* ≈ [1.1, 1.7] for which the network can attain reasonable firing rates. In the absence of inhibitory STDP, small changes of *β* either saturate firing rate at 320 Hz or neurons are quiescent, and no intermediate regime can be established.

Reasonable firing rates, i.e. rates that do not tend to either a maximum or zero, suggest a kind of balance, such as the balance of a critical network. To test the hypothesis that in our model this balance results from inhibitory plasticity, avalanche distributions were measured for a simulation in which inhibitory STDP was shut off after an initial period of time. That is, inhibitory synapses were frozen at whatever level they had reached at that point in the simulation, which was otherwise left to complete as usual. Avalanche distribution measurements for this simulation, following the same method as above, are shown in Fig. 9b. The data shows that after inhibitory STDP is turned off, avalanche distribution power-law exponents drift away from criticality. Other measures behave differently (Fig. 9c,d), highlighting the difference between balance and criticality. While the power law in avalanche size is lost and thus the system deviates from critical dynamics, the system remains stationary in its activity resulting in a maintained current balance close to zero and a branching ratio close to 1. These two measures, as said above, are necessary conditions for criticality; here that distinction is made clear.

#### Short and long-term plasticity

**Figure 10:**
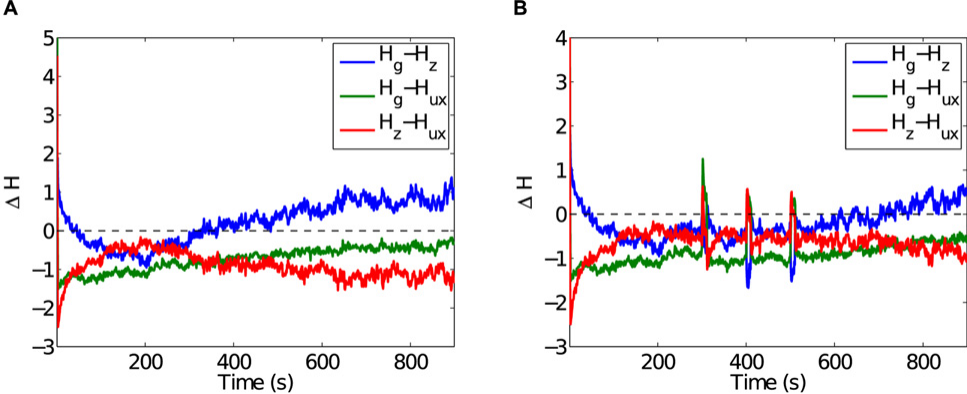
The difference in entropy Δ*H* between excitatory and inhibitory synaptic conductance changes due to STDP and change in synaptic efficacy due to STP. A) During early states, STDP is primarily responsible for changes to synaptic connection strength (Δ*H* > 0). Otherwise, STP appears more active (Δ*H* < 0) when the network is at rest. B) During strong perturbations, I-STDP becomes much more active than STP.

Synaptic plasticity appears to be responsible for a network’s ability to self-tune, both for maintenance of background activity and reaction to strong perturbations. Given two types of plasticity at work, we judged their relative roles by examining the Shannon entropy of their respective changes to synaptic weight (see Methods). Fig. 10 shows the entropy of synaptic efficacy, produced by STP, and conductance change, produced by STDP, as they change over time. A greater entropy suggests greater role of the corresponding plasticity mechanism. Our analysis shows that during early states of the simulation, STDP is primarily responsible for changes to synaptic connection strength. STP dominates when the network is at rest. During strong perturbations, I-STDP becomes more active than STP, further demonstrating the importance of I-STDP to maintain the network at criticality.

## Discussion

Here we show how synaptic plasticity allows neuronal networks to attain a number of desirable network dynamics and properties. First, it helps to produce PABA, persistent asynchronous background activity, which acts as a foundation for more specific behavior. Second, this foundational activity takes on characteristics of so-called critical networks. Lastly, synaptic plasticity enables the critical network, once established, to remain critical in the face of perturbations.

Spiking in the simulated network is only weakly correlated and irregular, shown by examining pairwise correlations between spike-trains and coefficient of variation within spike-trains. By restricting the range of ISIs considered, it can be concluded that spike bursts are responsible for a high coefficient of variation in otherwise Poisson-like spiking. Spiking with bursts can result from tonic input [35], but in this network bursts are favored by a *V*_*reset*_ voltage that is higher than *V*_*rest*_. After a spike, membrane voltages are decreased to *V*_*reset*_, which gives integration a head-start toward reaching the spiking threshold. This mechanism produces spikes on a time-scale near the refractory period of the neuron (see Fig. 12). Another possible source of bursts, most likely on a longer time-scale, is the action of STDP to create a bimodal distribution of synaptic strengths, which could take the place of manually increasing the *J* parameter in [35]. In any case, spike bursts contribute to high dimensional network activity, which increases input separability and dynamical memory capacity[35]. Avalanche size distributions, branching ratio, and correlated spike-interval fluctuations leading to DFA *α* > 0.5 show that the apparently irregular spiking activity is consistent with a critical network. A hallmark of self-organizing systems is a composition of relatively “dumb” units connected together and constrained by “interaction dominant dynamics” [36]. In the case of the simulated network presented above, the connection strength between units is altered by synaptic plasticity, effectively changing the network topology. The individual units, however, remain primarily unchanged. The structure of the network self-organizes such to combine uncorrelated units in a balanced way to produce network-level behavior that meets several criteria for criticality. This approach is different from other robust, balanced networks that rely on pre-constrained synaptic weights [37] and do not address the more subtle features of criticality, such as power-law avalanche sizes. In contrast, the network presented here adapts to perturbations with lasting changes to synaptic conductances (see Fig. 6) while maintaining the ability to self-tune towards a critical state.

The balance created is especially evident when following the reaction of the network to a single extra spike. Since the network dynamics are fully deterministic, we were able to follow two parallel realities for the network: one in which a particular spike occurred and one in which it didn’t. What results are two spike histories that begin to diverge exponentially, meaning that the network is sensitive to small changes in state. Since the network is finite, the spike-vector difference settles at a new value near the expected difference for two random spike-vectors. Balance in the network is also present at the level of excitatory and inhibitory currents. These currents are observed to balance each other, leaving the resultant current near zero. The level at which the currents balance is slightly excitatory, which makes sense for a spontaneously active network.

This last type of balance points towards the key mechanism for self-tuning. There are a limited number of ways that current into a neuron can be altered. In the present model, those ways are limited to changing synaptic conductance, via STDP, or changing synaptic efficacy, via STP.

It cannot be overstressed that both excitatory and inhibitory long-term plasticity are important, as it is the interplay between these two effects that results in the balanced, critical network achieved above. Networks without inhibitory STDP fail to reach this state for any of a large set of possible parameters. Even otherwise balanced networks without inhibitory STDP succumb to runaway positive feedback when stimulated by strong perturbations [38].

Fig. 9b shows a drift away from criticality after inhibitory STDP is switched off. A close inspection of the size distributions reveals that they contain a large amount of large avalanches, i.e. global bursts. Such global bursts tend to interrupt temporal correlations and spatial heterogeneity by globally depleting network resources. This interpretation is further supported by the finding that while a balance between excitatory and inhibitory current is maintained, the net positive current has increased making the network too excitable. Our simulations suggest that inhibitory STDP allows the network to respond rapidly enough to transient over-excitability to prevent resource depletions, which is crucial to maintain long-term temporal correlations in the system.

**Figure 11:**
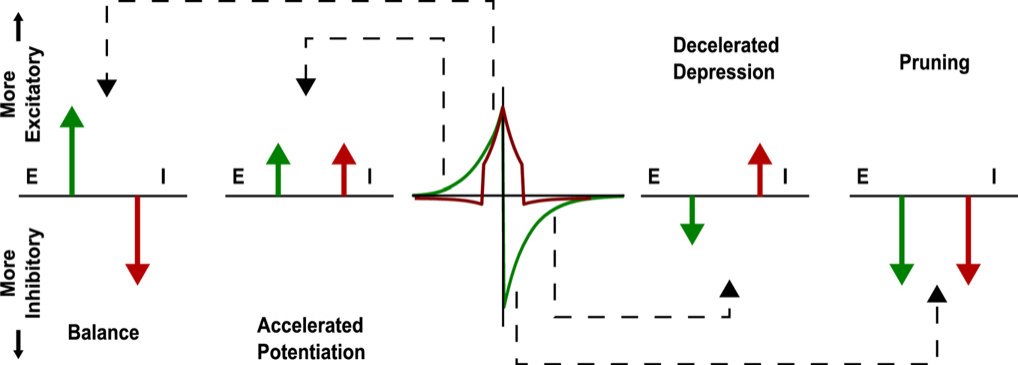
Inhibitory STDP interacts with excitatory STDP so as to favor balance among causal synaptic currents. Presyanptic and postsynaptic spikes can be causal or anti-causal to varying degrees. E-STDP and I-STDP combine to form four different behaviors: Balance, where excitation and inhibition both increase; Accelerated Potentiation, where excitation increases, but inhibition decreases; Decelerated Depression, where excitation and inhibition decrease, and Pruning, where excitation is decreased and inhibition is increased. Arrows pointing up indicate increasing excitatory effect, by either increasing excitatory weight or decreasing inhibitory weight. Arrows pointing down indicate increasing inhibitory effect, by either increasing inhibitory weight or decreasing excitatory weight. See Fig. 12 for details about STDP.

It is not only a practical matter that inhibitory STDP is required, but there are deep connections to self-organizing systems as well. Self-organization, especially self-organized criticality, is usually the result of two opposing effects, often some mutually-referring function of each other [39–42]. Here, excitatory and inhibitory STDP play these roles, and together produce various forms of compensatory feedback, depending on temporal differences between pre- and postsynaptic spikes [43, 44]. Fig. 11 shows a schematic description of how these two STDP functions combine to create a balanced network, in the hopes of attacking the “how” and “why” of the mechanisms involved. Further study on these points is required. We can discriminate roughly 4 different types of feedback depending on these temporal differences.

The inhibitory STDP function is symmetrical, supporting an increase in synaptic conductance, i.e. synaptic inhibition, for closely timed pre- and postsynaptic spikes regardless of their order. In contrast, the excitatory STDP function is anti-symmetric and biased towards depressing action. Together, averaged over the firing activity of a network, these two STDP functions combine such that along the Δ*t* = *t*_*pre*_ − *t*_*post*_ time-line, there are four qualitative regions: proximal causal and anti-causal, for those spikes that occur relatively close together, and distal causal and anti-causal, for those that occur farther apart. The difference in symmetry between E-STDP and I-STDP causes these regions to behave asymmetrically at the population level. In the *Balanced* regime, causal spikes that occur close to each other lead to a similar strong increase in excitation and inhibition. In the *Accelerated Potentiation* regime, where causal spikes occur at larger temporal distance, excitatory potentiation dominates whereas inhibitory STDP is absent or slightly negative. These leads to a temporal tightening of these causal spikes. In contrast, *Pruning* affects anti-causal spikes that are close in time. The decrease in excitatory drive and strong increase in inhibition for these spike pairs should loosen their temporal tightness and greatly reduce their probability of occurrence. Finally, in the *Decelerated Depression* regime, we encounter anti-causal spikes that are far-apart in time. In this regime E-STDP slightly dominates leading to reduction of the corresponding excitatory synapses, tempered by slight decreases in inhibition. The combined effect of the symmetry breaking is to foster balance among neurons that form networks of causal spiking, quickly reduce those that are strongly anti-causal, and maintain all other connections at a low, but non-vanishing level.

It is not surprising that inhibitory plasticity leads to balanced networks, as this phenomenon has been shown many times before [22, 43, 44]. The stabilizing nature of inhibitory plasticity is not the main issue here, but rather how that stability allows tuning towards critical spiking behavior. Furthermore, a stable network is not necessarily a critical one, as stability is necessary but not sufficient for criticality. Running the parameter search described in Code S1 on networks without plasticity results in some balanced networks that are not critical. Stability itself might come from or be enhanced by another homeostatic mechanism, such as synaptic scaling [45], however such mechanisms normally occur on much longer time-scales than the duration of the simulations presented above. Future investigations could focus on such questions.

The co-existence of PABA and self-tuning to criticality in our model is consistent with several experimental observations of the mammalian cortex. The cortex is spontaneously active both during development and in a fully developed cortex and this activity is asynchronous with low firing rates [28]. Neuronal avalanches are observed in animals [1], humans [7, 8], and *in vitro* cell cultures [4–6]. This suggests that natural neuronal activity exhibits PABA and has a tendency, on average, to operate close to its critical state.

While the model presented here takes many of its features from biological networks, and as just argued, shares important behaviors as well, there are still many differences. These differences reflect themselves in the model parameters that best show self-tuning. For instance, there is empirical evidence that the ratio of *τ*_*F*_ to *τ*_*AMPA*_ sits near 20 [46]. For this model, however, that ratio sits near 2. As such, this model is not intended to be a biological model in itself, but to encapsulate the driving dynamics for spiking networks that exhibit critical branching and avalanches. Networks of different size, topology, and with more or fewer biological features are expected to have different parameter sets for optimal self-tuning. Here, the qualitative dynamics of STDP and STP, and their role in critical networks, have been clarified. Furthermore, they have been quantified for a specific neuromorphic implementation of an E-I neuronal network using LIF neurons, two different STDP rules, and synaptic short-term depression. When considering neuromorphic systems in general, biological networks may be taken as a special case. Discovering which mechanisms in the general case are responsible for desired behavior, such as self-tuning to a critical state, provides insight into their presence in biological systems, but may also help to direct the design of large-scale artificial neuromorphic systems.

## Materials and Methods

### Network model overview

**Figure 12:**
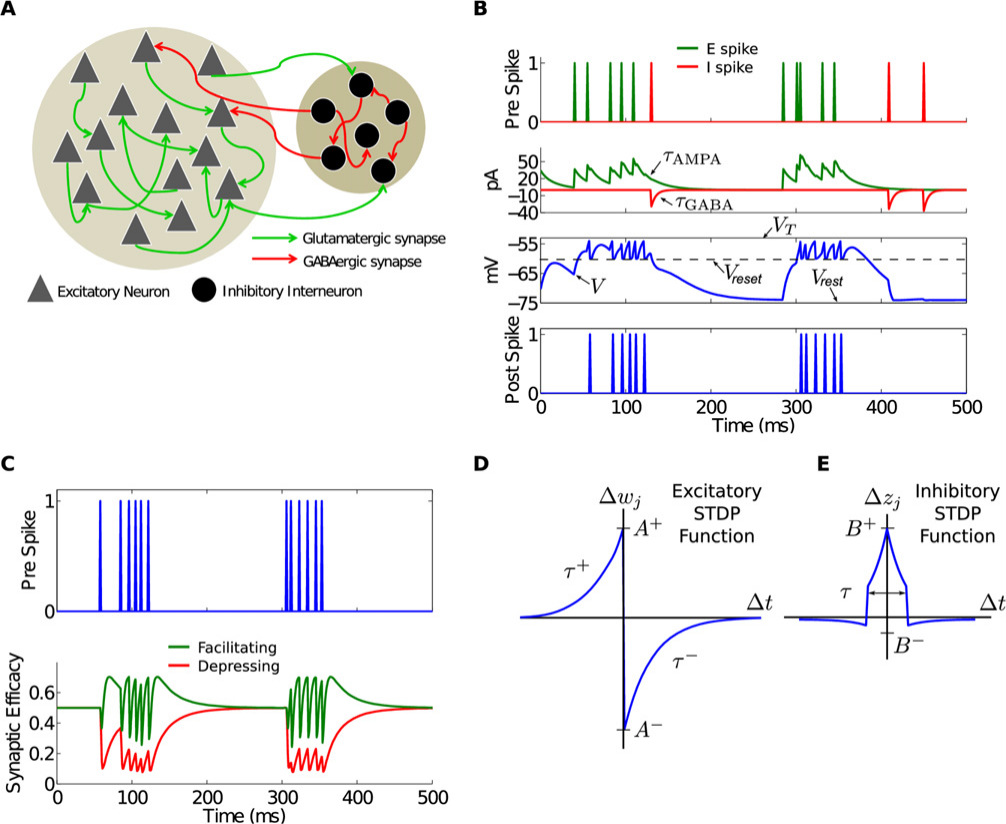
Model architecture and mechanisms. A) The *E*-*I* network model with two types of neurons: excitatory or *E* neurons and inhibitory or *I* neurons. There are also four types of synapses: *E* → *E*, *E* → *I*, *I* → *I*, *I* → *E*. B) The top two plots show presynaptic spikes from *E* and *I* neurons and the corresponding synaptic currents injected into a postsynaptic neuron. The synaptic currents are subject to AMPA and GABA receptor kinetics that are modeled using the two time constants *τ*_*AMPA*_ and *τ*_*GABA*_. The bottom two plots show the membrane voltage *V* and the corresponding spikes in the postsynaptic neuron modeled as an integrate-and-fire neuron that fires a spike when *V* > *V*_*T*_, returning to *V*_*reset*_ after each spike. C) The synaptic efficacy due to STP for the case of a single facilitating (*τ*_*F*_ > *τ*_*D*_) or a single depressing (*τ*_*D*_ > *τ*_*F*_) synapse is shown as a function of the presynaptic spike. In both cases, the efficacy relaxes back to *U* = 0.5 after the effect of the pre-spike has dissipated. D) The E-STDP is an asymmetric function of the timing difference ($\Delta t=t_{j}^{pre}-t_{j}^{post}$) between the pre- and postsynaptic spikes at neuron *j* and the corresponding change in synaptic conductance Δ*w*_*j*_ for *E* → *E* and *E* → *I* synapses. The four parameters (*A* ^+^, *A* ^−^, *τ* ^+^, *τ* ^−^) control the shape of the function and thus the amount of potentiation and depression. E) The I-STDP is a symmetric function of the timing difference ($\Delta t=t_{j}^{pre}-t_{j}^{post}$) between the pre- and postsynaptic spikes at neuron *j* and the corresponding change in synaptic conductance Δ*z*_*j*_ for *I* → *E* and *I* → *I* synapses. The three parameters (*B* ^+^, *B* ^−^, *τ*) control the shape of the function and thus the amount of potentiation and depression.

The recurrent neuronal network (Fig. 12a) consisted of 10,000 neurons composed of 8000 excitatory (*E*) neurons and 2000 inhibitory (*I*) neurons with a connection probability of 1%. Neurons were simulated as single compartments with leaky integrate-and-fire (LIF) dynamics. For this non-conservative network, synaptic input currents were modeled as exponentially decaying functions with a temporal time course approximating excitatory *α*-amino-3-hydroxy-5-methyl-4-isoxazole propionic acid (AMPA) and inhibitory gamma-aminobutyric acid (GABA) postsynaptic currents (Fig. 12b). Each combination of pre- and postsynaptic connections based on neuronal type were modeled, resulting in two excitatory (*E* → *E*, *E* → *I*) and two inhibitory (*I* → *I*, *I* → *E*) types of synapses. All synapses in the model continuously exhibited both short- and long-term plasticity. A notable aspect of our model is that its dynamics are completely deterministic and there is no explicit source for asynchronous or irregular firing such as external and irregular input or probabilistic synaptic transmission or action potential generation. Instead, PABA in the model emerged from intrinsic, deterministic dynamics. As shown, this deterministic design of the network allows for a detailed and precise analysis of the network response to extremely small changes such as the addition or removal of a single spike.

Two types of plasticity were implemented in the network. Short-term plasticity (STP), which transiently changes synaptic efficacy as a function of spike frequency in the presynaptic neuron (Fig. 12c) was simulated using a phenomenological model [47, 48] that combines short-term synaptic facilitation and depression. Long-term synaptic plasticity was implemented based on spike timing-dependent plasticity (STDP) rules at the level of network connectivity. In STDP, the temporal relationship between the arrival of synaptic input at a postsynaptic neuron and the action potential generation in the presynaptic neuron determines the magnitude and direction of the change at that particular synapse [22, 49–52]. We implemented STDP for excitatory synapses using the well established asymmetrical function (Fig. 12d). For inhibitory synapses (Fig. 12e), we used a recently reported symmetrical function [34, 53] (Fig. 12e).

#### Detailed Neuron Model

The leaky integrate-and-fire neuron (LIF) [54, 55] is used to model neuronal dynamics. Neurons were represented by a single compartment; no somatic, dendritic, or axonal specialization were modeled. In response to multiple input currents coming from excitatory and inhibitory presynaptic neurons in the sets *Pre*_*exc*_ and *Pre*_*inh*_, respectively, the membrane potential *V* for postsynaptic neuron *i* is determined by

$$\tau_{m}\frac{dV_{i}}{dt}=\left(V_{rest}-V_{i}\right)+\left(E_{ex}-V_{i}\right)\sum_{j\in Pre_{exc}}u_{ij}x_{ij}g_{w,ij}+\left(E_{inh}-V_{i}\right)\sum_{j\in Pre_{inh}}u_{ij}x_{ij}g_{z,ij}$$ (1)

When *V* reaches a threshold voltage *V*_*T*_ (Fig. 12b), the neuron fires a spike, and *V* is made equal to *V*_*reset*_, which was chosen to be 14 mV more positive than *V*_*rest*_. This basic model provides several control variables for the membrane voltage including conductances *g*_*w*_ and *g*_*z*_ for excitatory and inhibitory synaptic inputs respectively, synaptic efficacy *ux* (see STP Model), the membrane time constant *τ*_*m*_, the constant reversal potential for excitatory (*E*_*ex*_) and inhibitory (*E*_*inh*_) synaptic currents, and a fixed voltage threshold *V*_*T*_, at which the neuron fires a spike. Synaptic inputs to the neuron are modeled as conductances where a set of excitatory or inhibitory presynaptic spike times, *S*_*exc*_ or *S*_*inh*_ respectively, gives conductance dynamics,

$$\frac{dg_{w}}{dt}=\frac{-g_{w}}{\tau_{AMPA}}+w\sum_{s\in S_{exc}}\delta (t-s)$$ (2)

$$\frac{dg_{z}}{dt}=\frac{-g_{z}}{\tau_{GABA}}+z\sum_{s\in S_{inh}}\delta (t-s)$$ (3)

Here, the time constants *τ*_*AMPA*_ and *τ*_*GABA*_ approximate the average decay of AMPA and GABA currents respectively (Fig. 12b). The value of the excitatory and inhibitory synaptic conductances *w* and *z* are controlled by STDP. In all of our simulations, *τ*_*m*_ = 20 ms, *V*_*T*_ = −54 mV, *V*_*rest*_ = −74 mV, *V*_*reset*_ = −60 mV, *E*_*ex*_ = 0 mV, *E*_*inh*_ = −80 mV. The parameters *τ*_*AMPA*_ and *τ*_*GABA*_ were estimated using a parameter search process (Table 1) to establish PABA at low average firing rate. Simulations were based on fourth-order Runge-Kutta integration with a time step of 1 ms.

#### E-STDP Model

The E-STDP function modulates the change in excitatory synaptic conductance *w* based on the timing difference (*t*_*pre*_ − *t*_*post*_), or Δ*t*, between the spike times of corresponding pre- and postsynaptic neurons (Fig. 12c). The control parameters *τ* ^+^ = 20 ms and *τ* ^−^ = 20 ms determine the temporal window over which STDP is effective. The change in synaptic conductance is computed as

$$w_{new}=w_{old}+\Delta w$$ (4)

where

$$\Delta w=g_{exc}^{max}F\left(\Delta t\right)$$ (5)

and

$$F\left(\Delta t\right)=\left\{\begin{array}{cc} A^{+}\exp\frac{\Delta t}{\tau^{+}}, & \Delta t<0\\ -A^{-}\exp\frac{-\Delta t}{\tau^{-}}, & \Delta t\geq 0 \end{array}\right.$$ (6)

If $w_{new}>g_{exc}^{max}$, then $w_{new}=g_{exc}^{max}$. On the other hand, if *w*_*new*_ < 0, then *w*_*new*_ = 0. The factors *A* ^+^ and *β* = ∣*A* ^−^ *τ* ^−^∣/∣*A* ^+^ *τ* ^+^∣ control the increase or decrease in synaptic weight during learning [52], i.e. when STDP is active. The parameters *A* ^+^, *β*, and $g_{exc}^{max}$ were estimated using a parameter search process (Table 1). The initial excitatory synaptic conductance was set to 0.5 nS for all synapses.

#### I-STDP Model

The I-STDP function modulates the inhibitory synaptic conductance *z* [22, 34] based on the timing difference Δ*t* between the spike times of corresponding pre- and postsynaptic neurons (Fig. 12d). The synaptic conductance is computed as

$$z_{new}=z_{old}+\Delta z$$ (7)

The change Δ*z* = *B* ^+^ is governed by the following equations,

$$\Delta z=\left\{\begin{array}{cc} B^{+}\exp\frac{-|\Delta t|}{\tau }, & |\Delta t|\leq \tau \\ -B^{-}\exp\frac{-|\Delta t|}{\tau }, & |\Delta t|>\tau \end{array}\right.$$ (8)

If *z*_*new*_ < 0 then *z*_*new*_ = 0. On the other hand, if $z_{new}>g_{inh}^{max}$ then $z_{new}=g_{inh}^{max}$. The parameters in all of our simulations are set as: *B* ^+^ = 0.0015 nS, *B* ^−^ = 0.0003 nS, and *τ* = 10 ms. The parameter $g^{{max}_{inh}}$ was estimated during the parameter search process (Table 1). The initial inhibitory synaptic conductance was set to 0.5 nS for all synapses.

#### STP Model

STP was implemented using a well-established phenomenological model [47, 48], which modulates synaptic efficacy (*ux*) based on the dynamics of available resources *x* and the fraction *u* of these resources that are utilized by each presynaptic spike (Fig. 12e). The dynamics of these two parameters are described as

$$\frac{dx}{dt}=\frac{1-x}{\tau_{D}}-ux\delta (t-t_{sp})$$ (9)

$$\frac{du}{dt}=\frac{U-u}{\tau_{F}}+U(1-u)\delta (t-t_{sp})$$ (10)

where *U* is the baseline utility fraction set to 0.5 in all of our simulations and *δ* is the Dirac delta function, which models the presynaptic spike event *t*_*sp*_. The model reproduces the behavior of cortical synapses for both depressing (*τ*_*D*_ > *τ*_*F*_) and facilitating (*τ*_*F*_ > *τ*_*D*_) cases. The parameters *τ*_*D*_ and *τ*_*F*_ were estimated using a parameter search process (Table 1), resulting in a network with *τ*_*F*_ > *τ*_*D*_ for all synapses.

### Parameter Search

We first performed a parameter search (see Discussion for comments about self-tuning) to identify neuronal networks that exhibit PABA with relatively low firing rates when compared to other simulated networks of this type, e.g. [17].

The role of a parameter search in a model that claims to be self-tuning is worthy of further discussion. At first, any parameter search seems to be at odds with the concept of “self-tuning”. The search, however, is at a broad level that identifies networks that subsequently have the self-tuning property. That is, there are at least two levels at which manual parameter tuning might take place. Here, a parameter search is performed at one level, such that no manual tuning is needed at the other level. The benefit provided by this process is that the parameter tuning that is done is not problem specific, but yields networks that self-tune at the problem level.

While plasticity allows some amount of self-tuning, the parameters of a network must be within particular ranges for it to do so. To find reasonable values for the parameters in Table 1, we employed a biased random-walk searching algorithm. This search was primarily used to find networks with lower firing rates than would otherwise have occurred for random or arbitrary selection of parameters, as well as lower firing rates than other simulated networks of this type, e.g. [17]. As such, recent (*r*) and maximum (*r*_*max*_) firing rates were placed into a rate performance index, $R_{rate}=r^{2}+r_{max}^{2}$.

The eight parameters comprising the search space were: four parameters for STDP ($g_{exc}^{max}$, $g_{inh}^{max}$, *β*, *A* ^+^), two STP time constants for facilitation (*τ*_*F*_) and depression (*τ*_*D*_), and two receptor kinetic parameters *τ*_*AMPA*_ and *τ*_*GABA*_ for the neuron [48]. Networks with various combinations of these eight parameters received a 15 ms lasting initialization of Poisson-distributed spikes at 300 Hz to a randomly chosen set of 20 *E* neurons, and left to run for 900 s.

During the search, each parameter was perturbed by Gaussian noise with standard deviation equal to 2% of the parameter value. If such a perturbation resulted in an *R* less than the current best, those parameters became the new starting point for perturbations. Typically, a good region in the parameter space was found within 100 iterations. The parameter search algorithm is described in Code S1.

The first attribute to guide the search was to ensure that the network exhibited PABA. The criterion for determining if a network exhibited PABA was based on measuring the duration of time for which the average spiking activity of the network was non-zero after a brief external initialization with Poisson spikes. We selected networks that exhibited PABA for the full 900 s after initialization. We filtered the selected networks further on the basis of the second attribute that the average firing rate remained less than 35 Hz with a peak firing rate less than 100 Hz.

**Figure 13:**
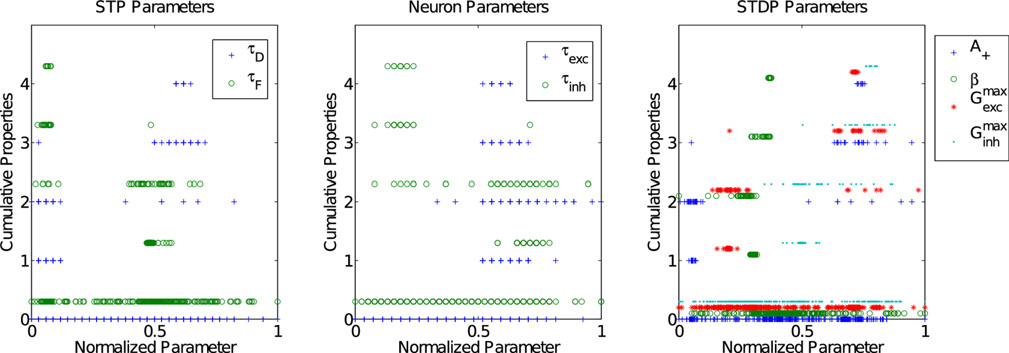
Number of desired properties (PABA and three measures of criticality) exhibited by simulations during the parameter search. Regions within a parameter’s normalized range are evident as showing more or fewer desired properties. Each property is normalized within its observed range to the interval [0, 1]. Vertically, each property is shifted slightly to aid visibility, so that a marker between two vertical axis tick-marks indicates a cumulative property count of the lower tick-mark. Each data-point represents a single network, simulated for 900 s with a particular set of parameters.

We then evaluated the resulting networks for criticality using three measures: a branching parameter *σ*, the avalanche power-law exponent *λ*, and the distribution of correlated fluctuations parameter *α*. We arrived at the final set of candidate networks that simultaneously have an average branching ratio *σ* ≈ 1, critical exponent *λ* ≈ −3/2 for avalanche activity and a correlated fluctuations parameter in the range 0.5 < *α* < 1. The results of these tests on each combination of parameters tried in the search are shown in Fig. 13.

### Criticality Measures on deterministic spike train sets

The output of a neural network exhibiting PABA is a relatively dense set of spike trains. The continuous nature of PABA makes analysis of spike propagation a non-trivial task. For instance, many avalanches, as described below, might overlap in a way that makes their detection difficult. In the paragraphs that follow, the techniques used to analyze such data are described. Unless otherwise specified, we used the following conventions: a neuron *i* is taken to generate a spike train *X*_*i*_(*n*), where *X*_*i*_(*n*) = 1 when there is a spike at time-step *n*, and *X*_*i*_(*n*) = 0 otherwise. Synaptic connections form a graph with matrix *G*, where *G*_*ij*_ = 1 if neuron *i* has a post-synaptic connection to neuron *j*. A particular spike is referenced as a tuple (*i*,*n*) implying *X*_*i*_(*n*) = 1. As a convenience, also let *X*(*n*) = {*i* : *X*_*i*_(*n*) = 1}, i.e. the set of neurons that spiked at time-step *n*.

#### Branching Ratio

The branching ratio of a spiking network measures the tendency of spikes to increase or decrease in number as they propagate through the network [4, 10]. In the first case, spiking activity grows over time, while in the latter it diminishes. Generally, the measure is a ratio of *post* spike activity to *pre* spike activity, such that if *post* activity is higher than *pre* activity, the branching ratio is greater than unity. Likewise, if *post* activity is lower than *pre* activity, the branching ratio is less than unity. Critical branching obtains when there is a balance between the two. Below, we define both a local and network level measure of branching ratio.

The local branching ratio at neuron *i* and time-step *n* was calculated for a given time window Δ and offset *ϕ* according to

$$\sigma_{i}(n)\;=\frac{\sum_{j}^{N}\overset{n+\phi +\Delta }{\underset{m=n+\phi +1}{\cup }}X_{j}(m)G_{ij}}{\sum_{j}^{N}\overset{n-\phi -1}{\underset{m=n-\phi -\Delta }{\cup }}X_{j}(m)G_{ji}}$$ (11)

$$\sigma (n)=\frac{\sum_{i}^{N}X_{i}(n)\sigma_{i}(n)}{\sum_{i}^{N}X_{i}(n)}$$ (12)

**Figure 14:**
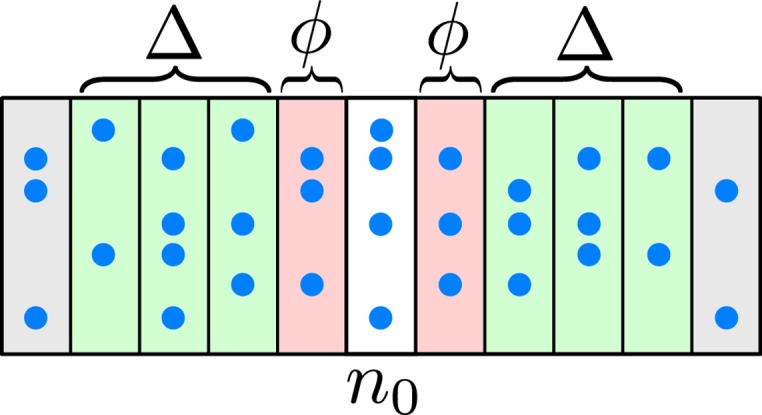
A schematic spike raster diagram consisting of a set of spikes over time. Rows and columns correspond to neurons and time-steps, respectively. Each blue dot represents a particular neuron spiking at a particular time-step *n*. Additionally, there are pre- (left of *n*_0_) and post- (right of *n*_0_) time windows and offsets. Time windows are offset by a value *ϕ* and have width Δ. Branching ratio requires both pre- and post-time-windows, while avalanche tracking requires only the pre-time-window.

That is, a ratio of the number of post-synaptic neurons that spiked within the post time-window to the number of pre-synaptic neurons that spiked within the pre time-window. See Fig. 14 for a depiction of time windows and offsets. The per-neuron ratio can be averaged over each neuron that spiked at time-step *n* to define a whole-network measurement of local branching.

A network-level branching ratio can be defined slightly differently by computing the ratio for the sum of all pre-synaptic and post-synaptic spikes in the network at time-step *n*.

$$\sigma (n)=\frac{\sum_{i}^{N}X_{i}(n)\sum_{j}^{N}\overset{n+\phi +\Delta }{\underset{m=n+\phi +1}{\cup }}X_{j}(m)G_{ij}}{\sum_{i}^{N}X_{i}(n)\sum_{j}^{N}\overset{n-\phi -1}{\underset{m=n-\phi -\Delta }{\cup }}X_{j}(m)G_{ji}}$$ (13)

This measurement is more representative of the network branching ratio, as it takes into account shared connections more readily. For instance, if spiking neurons *a* and *b* both have *c* as a post-synaptic connection, then a subsequent spike from *c* is counted towards the post-count of both *a* and *b*. This is the method used in the presented analysis, with *ϕ* = 1 due to a 1 time-step delay in the network and Δ = 3. Both methods show branching ratios near unity when the network is exhibiting PABA.

While a branching ratio near unity is supporting evidence of criticality, it is not specific to it. Any self-maintaining deterministic network is expected to have this feature. As such, this measurement should be taken together with the other measures of criticality that follow.

#### Avalanche distributions

An avalanche is a sequence of causally contiguous spikes. If a pre-synaptic spike causes a subsequent, post-synaptic spike, those spikes are said to be members of the same avalanche. A spike that is not caused by a pre-synaptic spike, instead caused by either spontaneous activity or some other external influence, is the start of a new avalanche. In a complex network that contains decay times and delays the notion of a causal chain loses some meaning. As such, it is necessary to describe what counts as a contributing cause.

In the present analysis, a spike at time-step *n* is said to be the product of *all* pre-synaptic spikes that occur within a time window prior to *n* with width Δ and offset *ϕ* (see Fig. 14). That is, pre-synaptic spikes that fall between *n* − *ϕ* − Δ and *n* − *ϕ* − 1 inclusive, are contributing spikes. Alternatives exist to this assumption, such as picking a single pre-synaptic spike or choosing a set using a synaptic strength threshold. It is not straightforward to say for certain whether a small current did or did not contribute to a spike. As such, we choose to assume that any pre-synaptic spike may count as causal. The choice of Δ*n* and *ϕ* depend on the nature of decays, delays, and other non-linear effects in the network. Decay and refractory times determine a meaningful Δ, while delay times determine *ϕ*. The effect of altering parameters *ϕ* and Δ has not yet been characterized analytically. Qualitatively, however, there are limits to the ranges that make sense for a given network and that will produce reasonably representative avalanche size distributions. For instance, if Δ is too large, then the time window can overlap causal pre-post relationships. Breaking the causal links between time windows destroys the ability to track avalanches, and so results in many avalanches of very small size. Likewise, choosing a *ϕ* that is too large will skip over causal links, resulting in the same kind of degenerate avalanche size distribution. On the other end, choosing a small Δ will discount the effects of leaky currents and underestimate causal responsibility. Finally, a small *ϕ* includes spikes that are outside of the “light-cone” of the network, and couldn’t be causal.

Taking the *k*th avalanche as a set of spikes *A*_*k*_, then spike (*i*, *n*) ∈ *A*_*k*_ if ∃*s* ∈ [*n* − *ϕ* − Δ, *n* − *ϕ* − 1] ((*j*, *s*) ∈ *A*_*k*_ ∧ *G*_*ji*_ = 1). Otherwise, it is the beginning of a new avalanche and *A*_*k*_ contains only (*i*,*n*). The distribution of avalanche sizes is then taken to be the distribution of set cardinalities.

It is possible to use this same procedure in order to examine a subset of neurons. For instance, neurons can be sampled from the network with some probability *p*, 12.5% in our analyses. This is meant to be analogous to measuring activation in a patch of *in vivo* tissue [56]. This assertion is further examined below.

Connectivity is initially random. That is, for all nodes *v* ∈ *V*, edges are created uniformly with probability *c*. This is equivalent to an Erdős-Rényi graph *G*(*N*, *c*) where *N* = #*V*. Otherwise, *G*(*N*, *c*) is known as *G*(*V*, *E*) with nodes *V* and edges *E*. Random sampling selects nodes *w* ∈ *W* ⊆ *V* with probability *p*. A sampled subgraph can then be defined as *H*(*W*, *E* ^′^), where *E* ^′^ = {(*x*, *y*) : (*x*, *y*) ∈ *E* ∧ *x* ∈ *W* ∧ *y* ∈ *W*}. That is, the subgraph contains the sampled nodes and all and only those edges that exist between sampled nodes.

The analogy to *in vivo* sampling is motivated by the ability to take any such graph *H* and to arbitrarily distribute its nodes *W* along a region of a manifold, such as a surface patch of cortical tissue. It is prudent to note that the degree distribution of *H* is reduced from that of the full graph *G*. For an arc (*x*, *y*), *x* ∈ *H*, *P*(*y* ∈ *H*) = *cp* and *P*(*y* ∉ *H*) = *c*(1 − *p*). If the expected in and out degree of *G* are both *cN*, the reduced out degree would then be *cpN*. Empirically, the avalanche distributions obtained via sampling do not show peaks at multiples of the sample size, suggesting that the sample is large enough compared to large avalanches [57], however sub-critical deviations at large avalanche sizes might still be a result of sampling (see Results). The implementation of this process is described in Code S2. The method described above produces a discrete distribution of avalanche sizes. In order to estimate a scaling exponent, the distribution, without binning, was fit using linear regression.

#### Detrended Fluctuation Analysis

Detrended Fluctuation Analysis [58] (DFA) aims to find the functional relationship between fluctuations and time scale. A spike train *X*_*i*_(*n*) has an equivalent representation of inter-spike-intervals, where *Y*_*i*_(*s*) is the time interval between spike *s* and spike *s*+1 from neuron *i*. To determine the DFA fluctuation function, *Y*_*i*_(*s*) is first centered and integrated,

$${\bar{Y}}_{i}=\frac{1}{N_{i}}\sum_{s=1}^{N_{i}}Y_{i}(s)$$ (14)

$$y_{i}(s)=\sum_{s=1}^{N_{i}}(Y_{i}(s)-{\bar{Y}}_{i})$$ (15)

where *N*_*i*_ is the number of spike intervals in *Y*_*i*_.

The integrated series is then split into *M* equal segments *z*_*m*_ of length *l* such that *N*_*i*_ = *Ml*. From this point, the index *i* for a particular neuron is assumed. Each segment *m* is individually detrended by removing some fit of the segment, *ẑ*_*m*_, such as a least squares approximation. Typically this is a first order linear regression. Fluctuations are measured at the segment level and averaged to achieve a fluctuation measurement at that scale.

$$F^{2}\left(l\right)=\frac{1}{M}\overset{M}{\underset{m=1}{\sum }}{\left(z_{m}-{\hat{z}}_{m}\right)}^{2}$$ (16)

For a range of possible segment scales, *F* ^2^(*l*) will show how the measure of fluctuation varies with those scales. It has been shown [59] that processes with algebraically decaying autocorrelation functions, as opposed to exponentially decaying, have a power law fluctuation function

$$F^{2}\left(l\right)\propto l^{\alpha },$$ (17)

where *α* is said to be the *scaling exponent*. If the underlying fluctuations conform to a power law with exponent *α*, that exponent can be estimated using a linear regression in log-log coordinates. Note, however, that there are significant caveats when doing so [60, 61].

### Entropy of weights and synaptic efficacy

Each neuron in the network receives input from many other neurons, each with an associated synapse. In turn, each synapse maintains what amounts to a connection strength or weight. These weights change over time due to the effects of STDP. Synaptic efficacy, on the other hand, is an instantaneous property affected by STP. Both of these changing synaptic parameters affect the strength of connection between two neurons, but they do so in different ways and might be more or less active at different times. One way to capture this difference is to examine the entropy of each measure over time.

For neuron *i* and synapse *j*, there are synaptic weights *g* and synaptic efficacies *ux*. Entropy over a time window Δ*t* was computed for each neuron at each time-step *n*Δ*t*. For quantity *y*, which can either be *g* or *ux*, the quantity’s distribution within the time-step must be determined

$$h_{k}(t)\;=\int_{t}^{t+\Delta t}\int_{b_{k}}^{b_{k+1}}\delta (x-y_{j}(\tau ))dxd\tau$$ (18)

$$p_{k}(t)\;=\frac{h_{k}}{\sum_{k}h_{k}}$$ (19)

so that *h*_*k*_(*t*) is the number of times *b*_*k*_ ≤ *y*_*j*_ < *b*_*k*+1_ within the time-window beginning at *t*, which can be expressed as a probability *p*_*k*_(*t*). Given a probability distribution, it is straightforward to compute the Shannon entropy for the quantity *y* over time,

$$H_{y}(t)=-\sum_{k=1}^{K}p_{k}(t)\log_{2}p_{k}(t)$$ (20)

where *K* is the total number of bins. We used this measure of Shannon entropy to track the respective contributions of STDP and STP by computing a difference in entropy Δ*H* as

$$\Delta H\left(t\right)=H_{g}\left(t\right)-H_{ux}\left(t\right)$$ (21)

where *H*_*g*_ represents entropy due to STDP, while *H*_*ux*_ represents entropy due to STP. Furthermore, we may reserve *H*_*g*_ for excitatory STDP and additionally take the difference of inhibitory STDP and STP factor, *H*_*z*_(*t*) − *H*_*ux*_, or excitatory and inhibitory STDP, *H*_*g*_(*t*) − *H*_*z*_(*t*).

### Weight analyses

Synaptic weights were compared between pulsed and non-pulsed conditions using a simple mean square error (MSE). That is, for a weight vector *w*_1_, containing the weights of all of the synapses in the first network, and *w*_2_, containing the same for the second, the distance between the weights was calculated using

$$MSE=\frac{∥w_{1}-w_{2}{∥}^{2}}{N}$$ (22)

where *N* is the total number of synapses.

A second way to measure synaptic weight evolution was to identify times at which individual synapses transition from high weight to low weight, or *vice versa*. High weight for neuron *i* was defined to be *g*_*i*_ > 0.9, and low weight *g*_*i*_ < 0.1. Transitioning from one regime to the other is termed a *flip*, and represents a particular synaptic connection being turned on or off. To determine flipping points, each synaptic weight was tracked over time at a resolution of 1000 ms. Time points at which a synapse left the high or low regime were recorded. If at a subsequent time a synapse entered the other regime, a flip was recorded at that time.

### Deterministic component estimation

Given a first-order dynamical system,

$$\dot{x}\left(t\right)=h\left(x\right)+\Gamma \left(t\right)$$ (23)

*h*(*x*) and zero-mean stochastic component Γ(*t*), it is possible to recover the deterministic component using an ensemble average [30, 31]

$$\langle \dot{x}\left(t\right)\rangle \;=\;h\left(x\right)$$ (24)

Approximation of the ensemble average from a discrete time-series *y*, sampled from dynamical system *x*, can be achieved by binning the range of *y* into bins *b*_0_,..,*b*_*n*_. For each data point *y*_*m*_ in bin *k*, *b*_*k*_ ≤ *y*_*m*_ < *b*_*k*+1_, take the mean Δ*y*(*k*) = ⟨*y*_*m*+1_ − *y*_*m*_⟩. The resulting mapping from *k* to Δ*y* approximates *h*(*x*), the deterministic component of *x*. From this point, zero-crossings of Δ*y* denote stable (negative slope) or unstable (positive slope) fixed points in the dynamics of *x*. This interpretation cannot be made with confidence if the system undergoes bifurcations (a change in the type or number of fixed points) during the analyzed time or contains more dimensions than are represented by *y*.

### Primate Recordings and Comparison

Ongoing LFP (1–100Hz) and extracellular spike (300 - 3000 Hz; offline sorted; Plexon Offline sorter; PCA based) activities were recorded using microelectrode arrays (BlackRock; 400 mm inter-electrode distance; shank length: 1 mm) chronically implanted in the premotor cortex of two monkeys (Macaca Mulatta), sitting in a primate chair, alert, but not engaging in any task. Measurements were taken for 20–30 minutes. The power law in avalanche sizes for this resting activity was published in [27].

## Supporting Information

S1 Code Parameter search algorithm. This algorithm uses biased random walk, similar to a simplified Metropolis algorithm, in order to find a collection of parameter sets that result in PABA.
(TXT)

S2 Code Algorithm for tracking overlapping avalanches in a set of spike trains. Every presynaptic spike is assumed to be possibly causal, and therefore part of an avalanche. A single spike can be part of multiple avalanches, in order to track many overlapping avalanches.
(TXT)
